# Mapping of mosquito breeding sites in malaria endemic areas in Pos Lenjang, Kuala Lipis, Pahang, Malaysia

**DOI:** 10.1186/1475-2875-10-361

**Published:** 2011-12-13

**Authors:** Rohani Ahmad, Wan NWM Ali, Zurainee M Nor, Zamree Ismail, Azahari A Hadi, Mohd N Ibrahim, Lee H Lim

**Affiliations:** 1 Medical Entomology Unit, Infectious Disease Research Centre, Institute for Medical Research, 50588 Kuala Lumpur, Malaysia; 2 Department of Parasitology, Faculty of Medicine, University of Malaya, 50603 Kuala Lumpur, Malaysia

## Abstract

**Background:**

The application of the Geographic Information Systems (GIS) to the study of vector transmitted diseases considerably improves the management of the information obtained from the field survey and facilitates the study of the distribution patterns of the vector species.

**Methods:**

As part of a study to assess remote sensing data as a tool for vector mapping, geographical features like rivers, small streams, forest, roads and residential area were digitized from the satellite images and overlaid with entomological data. Map of larval breeding habitats distribution and map of malaria transmission risk area were developed using a combination of field data, satellite image analysis and GIS technique. All digital data in the GIS were displayed in the WGS 1984 coordinate system. Six occasions of larval surveillance were also conducted to determine the species of mosquitoes, their characteristics and the abundance of habitats.

**Results:**

Larval survey studies showed that anopheline and culicine larvae were collected and mapped from 79 and 67 breeding sites respectively. Breeding habitats were located at 100-400 m from human settlement. Map of villages with 400 m buffer zone visualizes that more than 80% of *Anopheles maculatus s.s*. immature habitats were found within the buffer zone.

**Conclusions:**

This study amplifies the need for a broadening of the GIS approach which is emphasized with the aim of rejuvenating the dynamic aspect of entomological studies in Malaysia. In fact, the use of such basic GIS platforms promote a more rational basis for strategic planning and management in the control of endemic diseases at the national level.

## Background

Malaria is one of the most widespread diseases in the world. It is endemic throughout the tropical and subtropical regions of the world [1,2]. Malaria remains a public health problem in Malaysia, especially in the state of Sabah, Sarawak and in the interior central regions of Peninsular Malaysia where Perak, Pahang and Kelantan share their borders and where the population is made up of aborigines [3]. In recent years however, there are also reports of malaria outbreak occurring in states previously known to be free of malaria, such as Penang and Negeri Sembilan in 2008 [4].

Malaysia is currently in the pre-elimination stage and aiming towards malaria elimination by 2015 [5]. The effort to combat malaria started in 1967 with the launching of Malaria Eradication Programme (MEP) in Peninsular Malaysia. In 1980, MEP objectives were further improved towards employing a more realistic approach that is, towards controlling the disease, known as the Malaria Control Programme (MCP) [6]. MCP was extended to Sabah and Sarawak in 1986. By then, the programme was reorganized to include other vector borne diseases namely; dengue, filariasis, typhus, Japanese encephalitis, yellow fever and plaque, and came to be known as Vector Borne Diseases Control Programme (VBDCP).

To date, studies of anthropophilic *Anopheles *mosquitoes are scarce, especially in the interior hilly areas of Peninsular Malaysia where malaria is known to exist. There are also several unique challenges that need to be addressed when dealing with area having low endemic malaria [7]. Information such as knowledge of the composition and the biting habits of anopheline mosquitoes associated with malarious areas are important and need to be considered for any control programmes to be executed successfully [8].

Traditionally, the control of disease-bearing vectors relies heavily on the extensive use of chemical insecticides. Those chemicals are, to a certain extent, quite successful in curbing the disease concerned. However, the widespread use of chemicals is highly stressful on the environment. Besides, it also causes undesirable side effects on the complex tropical ecosystem and may affect a wide spectrum of non-targeted organism [9]. Meanwhile, effective control of malaria through vector management requires information on the distribution and abundance of vectors in the targeted area [10].

A better approach to assess the risk of malaria transmission by malaria vector control is through the study of anopheline larvae [11]. Source reduction through modification of larval habitats is the key to malaria eradication efforts. It is conceivable that appropriate management of larval habitat in this country can suppress vector and malaria transmission. However, anopheline larval ecology is limited, and the knowledge is insufficient to achieve effective vector control through the means of larval control. Minakawa *et al *[12] said that one major reason for the lack of ecologic studies on anopheline larvae is the difficulty involved with larval sampling from aquatic habitats in the field, particularly when many larval habitats are not permanent.

Instead of a traditional ground-based approach to vector surveillance, new tools such as the geographic positioning system (GPS) and geographic information system (GIS) technologies are now available for mapping larval habitats [12]. They are useful tools to forecast malaria transmission by tracing potential breeding habitats for malaria vectors from changes in the environment detected by the satellite imagery observation. Certain environmental changes like deforestation and vegetation clearance for crop plantations may lead to an increase abundance of mosquito larval habitats [7,13]. GIS has been used to map malaria vectors [14], vector habitats [12] and infections [15]. It has also been used in the management and control of malaria [16,17], to measure the effect of access to malaria treatment and to evaluate the effects of intervention strategies [18]. The application of GIS also considerably improves the management of information obtained from field surveys and facilitates a study of the distribution patterns of vector species. Thus, it could be applied to vector control measure by environmental management method for man-mosquito contact reduction.

This study aims to optimize the use of Remote Sensing (RS) and GIS technologies in malaria control programme by examining the spatial distribution of vectors in malaria endemic areas and determining the correlation between environmental variables and the distribution of larval in the breeding habitats. It is hoped that the information gathered from this study will help to broaden the understanding with regards to the geography, biology and ecology of mosquito breeding sites, and thus effective and efficient larval control measures can be applied.

## Methods

### Study site

Study was carried out in Pos Lenjang (N4°15.413' E101°32.843), Kuala Lipis, Pahang which is located about 240 km east of Kuala Lumpur. Pos Lenjang consists of 17 Orang Asli (aborigine) villages with capacity of 1,470 people and 310 houses. The houses in Lenjang are made of a mixture of bamboo, trees bark and wood. The aborigine in Pos Lenjang comprises the Semai ethnic group. The villages in Pos Lenjang are carved out of a secondary forest situated on hilly terrain, with most villages being riverine. Most of these villages are in remote areas and accessible only by four-wheel drive vehicle, while others can only be reached by foot. It took 3 h on a logging track to reach Pos Lenjang. The houses are scattered about in the area, usually in the clearing of the foothills. The Pos Lenjang population continues to live in a semi-nomadic lifestyle. Villages in Pos Lenjang are not static as houses and sometimes whole villages may be relocated to remain within convenient walking distance of garden crops which are periodically moved to different places to reflect agricultural practices within traditional land tenure areas. Epidemiology of malaria in Kuala Lipis are influenced by crop plantation, fruit season and hunting activities. The study areas were selected based on available epidemiological records (five consecutive years of high malaria cases in Peninsular Malaysia) and *Anopheles maculatus *is known to be the principal vector of human malaria in Kuala Lipis [19]. Malaria control program in Pos Lenjang is managed by Department of Arborigional Affairs, Ministry of Home Affairs Malaysia.

### Larval surveillance

Larval collections were done in order to determine the types and abundance of habitats where potential vectors exist. Larval collection and site mapping were conducted every alternate month on six occasions between Mac 2009 and December 2009. All possible bodies of water were sampled within and surrounding areas of the 17 villages and along all the rivers in Pos Lenjang. All 17 villages are situated near the rivers and these rivers are connected to one another. All potential habitats were inspected systematically for the presence of mosquito larvae. When mosquito larvae were present, a standard mosquito dipper was used to collect the larvae by lowering it gently into the water. The water was poured in 30 × 15 cm plastic tray and carefully observed for the presence of mosquito larvae. Then, larvae were collected alive by means of a pipette and transferred to a labeled bottle. Each bottle was covered tightly to prevent spillage and to ensure that the larvae collected remained alive and undamaged as they were transported to the insectarium. The larvae collected were reared in the insectarium in white plastic tray on a diet of ground ox liver.

### Identification of mosquito larvae collected in the survey

Mosquito larvae collected in the survey were reared to adults [11] and adults were identified by species using standard taxonomic keys [20-22]. 93.6-96.2% of the larvae collected survived to adults and the rest of the larvae (3.8-6.6%) were not identified and discarded.

### Mapping of mosquito population

The coordinates of habitat containing mosquito larvae were marked using a hand-held Geographic Positioning System (GPS), [Garmin GPSMAP^® ^60CSx] and processed with MapSource^® ^software [23]. Raster image (dated 11 November 2009) of Pos Lenjang and surrounding area generated from Google search engine were used. Features like water body, forest, vegetation areas, roads and residential area were extracted and digitized from the raster image as feature layers. Coordinates of habitat containing mosquito larvae were later integrated to a GIS database using software ArcGIS 9.3 [24] to quantify spatial heterogeneity in the associated area. Raster images of the site were overlaid with feature layers and entomological data in a GIS database. All digital data in the GIS were displayed in the WGS 1984 Coordinate system.

## Results

### Larval surveillance

A total of 291 of anopheline and 164 of culicine mosquito larvae were collected from 120 larval breeding habitats. Immature *Anopheles *mosquitoes were taxonomically identified to *Anopheles maculatus s.s*. (43.3%) and *Anopheles macarthuri *(46.7%). Culicine mosquitoes were taxonomically identified to *Aedes butleri *(7.32%)*, Aedes (Finlaya) macfarlanei *(7.32%)*, Aedes pseudoalbopictus *(11.59%)*, Armigeres *sp. (12.20%)*, Culex fuscanus *(8.54%)*, Culex mimeticus sg*. (8.54%)*, Culex nigropunctatus *(23.17%)*, Tripteroides *sp. (6.10%) and *Uranotaenia *sp. (15.24%).

### Breeding type

This study characterized and identified key environmental factors for mosquito breeding habitats. A total of 120 breeding habitats were sampled and classified into nine groups combining three substrate types and three habitat types namely clear ground pool, cloudy ground pool, muddy ground pool, clear rock pool, cloudy rock pool, muddy rock pool, clear water pocket, cloudy water pocket and muddy water pocket. Muddy water refers to a muddy area that holds water. Cloudy water refers to an area with rocks or sand that holds water that contains debris or dead leaves. The water in the breeding sites was from the rainfall or rivers.

Table 1 shows the distribution for anopheline and culicine mosquitoes out of 120 habitats sampled. Anopheline larvae were found in 79 habitats, of which 53 (44.17%) of these had only anopheline. Culicine larvae were found in 67 habitats, and 41 (34.17%) of these habitats had only culicine. Both anopheline and culicine larvae were found in 26 habitats (21.67%), suggesting that the mosquito larvae from the subfamilies culicinae and anopheline coexisted in some of the habitats surveyed. Only two *Anopheles *species (*Anopheles maculatus s.s*. and *Anopheles macarthuri*) were found during the survey and these species coexisted in 18 breeding habitats. The highest number of breeding site for anopheline mosquito larvae was clear rock pool. The most common breeding site for culicine larvae was cloudy rock pool. The most common breeding site where anopheline and culicine mosquito larvae co-existed was clear rock pool.

**Table 1:** Distribution of anopheline and culicine mosquito larvae from the total of 120 aquatic habitats sampled in Pos Lenjang, Kuala Lipis, Pahang

		**Larval habitat group**								
		
	**Substrate type**	**Clear**	**Cloudy**	**Muddy**	**Clear**	**Cloudy**	**Muddy**	**Clear**	**Cloudy**	**Muddy**
	
	**Habitat type**	**Ground pool**			**Rock pool**			**Water pocket**		
	**Number of habitats**									
**Anopheline vs. Culicine**										
Presence of anopheline larvae only	53 (44.17%)	2	0	1	20	2	7	14	0	7
Presence of culicine larvae only	41 (34.17%)	3	2	1	7	8	6	5	4	5
Presence both anopheline and culicine larvae	26 (21.67%)	0	0	0	8	0	2	5	5	6
Total	120 (100%)	5	2	2	35	10	15	24	9	18
***Anopheles***										
Presence of *An. maculatus *larvae only	14 (17.72%)	0	0	0	7	0	1	5	0	1
Presence of *An. macarthuri *larvae only	47 (59.49%)	1	0	1	17	2	6	9	2	9
Presence of both *An. maculatus *and *An. macarthuri *larvae	18 (22.78%)	1	0	0	5	0	1	5	3	3
Total	79 (100%)	2	0	1	29	2	9	19	5	13

*An. maculatus s.s*. was present in six out of nine habitat groups. *An. maculatus s.s*. was not found in cloudy ground pool, muddy ground pool and cloudy rock pool. The most common larval habitat for *An. maculatus s.s*. were clear rock pool (12), followed by clear water pocket (10), muddy water pocket (4) and muddy rock pool (2). It was found that 82.28% of anopheline breeding sites were positive with *An. macarthuri*, which was found in all the group types except cloudy ground pool.

### Distance to nearest house

**Figure 1:**
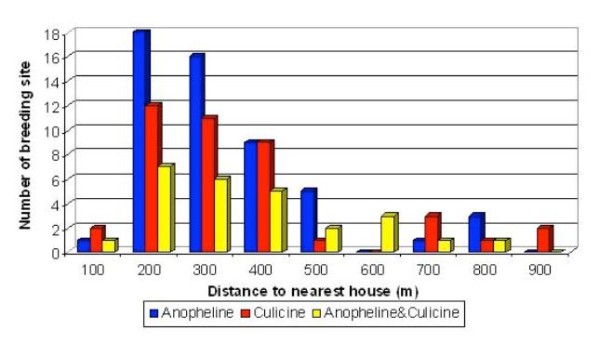
**Distribution of anopheline and culicine larvae in habitats with different distance from nearby village**.

The distribution of anopheline and culicine larvae in habitat with different distance from nearby village is shown in Figure 1. Highest numbers of breeding habitat were recorded at the area with distance between 200 m to 400 m from the nearest house. This proved that after obtaining blood meal required for the egg development at nearby villages, the mosquito may fly as far as 400 m for suitable habitat to breed. The range of 200 m included 18 habitats with only anopheline, 12 habitats with only culicine and seven habitats having anopheline and culicine. The range of 300 m included 16 habitats having only anopheline, 11 habitats with only culicine and six habitats having both anopheline and culicine. The range of 400 m included nine habitats inhabitated by only anopheline, nine habitats having only culicine and five habitats contained anopheline and culicine. Very few habitats were found in the 100 m distance range. This could be due to the activities conducted by the settlers such as deforestation for settlement, crop plantation' bathing and washing have resulted in substantial disturbance to any available potential habitats. Only culicine were found breeding in area more than 800 m away from the village.

**Figure 2:**
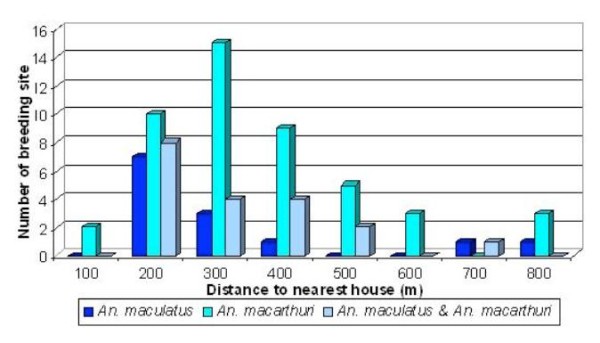
**Distribution of *Anopheles *species in habitats with different distance from nearby village**.

Figure 2 shows distribution of anopheline species in habitat with different distance from nearby village. This finding clearly showed that between the two *Anopheles *species, *An. maculatus s.s*. prefers to breed in area closer to houses especially within 200 m range compared to *An. macarthuri*. *An. maculatus s.s*. seemed to be more selective in choosing their habitat as indicated by the decrease in habitat number as the distance increased. The larval survey results showed that, there are many potential breeding sites along the rivers in the study areas but only those near to human settlement (200 m-400 m) were found positive for *An. maculatus s.s*. *An. macarthuri *however were found to be more flexible in choosing their habitat in relation to distance from houses because they were found breeding in area covering wider range (100 m-800 m) although 200 m-400 m range seemed to be most favourable distances.

### Mapping of larval habitat distribution

**Figure 3:**
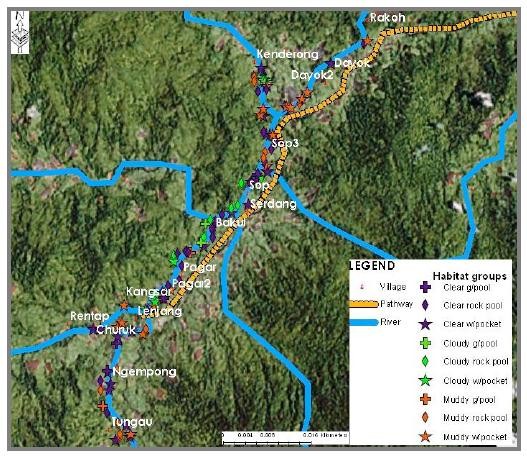
**Distribution of nine larval habitat groups in Pos Lenjang**.

**Figure 4:**
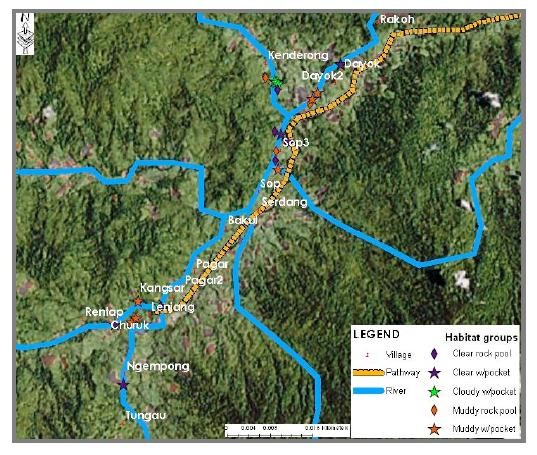
**Distribution of breeding habitat groups for anopheline plus culicine larvae in Pos Lenjang**.

**Figure 5:**
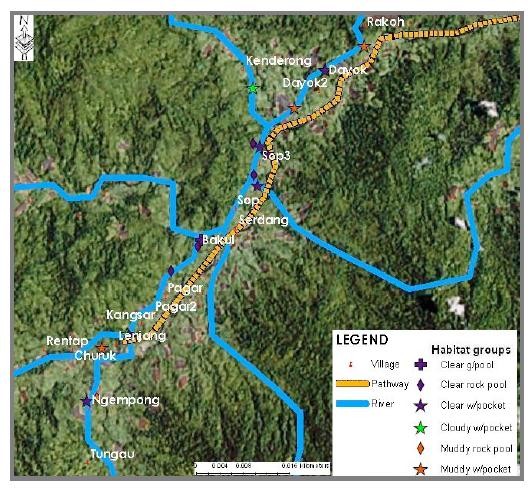
**Distribution of breeding habitat groups for *An. maculatus *plus *An.macarthuri *larvae in Pos Lenjang**.

**Figure 6:**
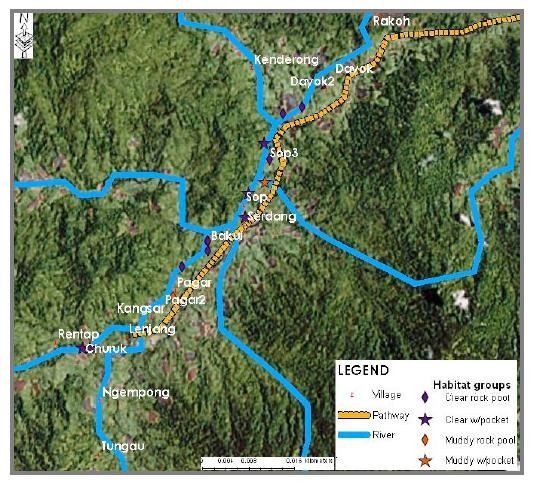
**Distribution of breeding habitat groups for *An. maculatus *larvae in Pos Lenjang**.

This study has provided us with data that allow us to produce several maps that illustrated the distribution of larval breeding habitat in Pos Lenjang, Kuala Lipis, Pahang. Figure 3 is a map showing the distribution of the nine larval habitat groups found in Pos Lenjang. All these habitats were located next to river margin that passed through Pos Lenjang. As such, none of the breeding sites was found in the river margins far away from the villages. Figure 4 is a map that shows the distribution of the larval breeding habitat groups according to mosquito's genera: anopheline plus culicine. A map is also produced visualising *Anopheles *species larvae identified for each habitat. Figure 5 visualized the distribution of villages and the habitat groups according to *An. maculatus s.s*. together with *An. macarthuri *and Figure 6 visualized the distribution of villages and the habitat groups according to *An. maculatus s.s*. only.

### Malaria transmission risk area in Pos Lenjang

**Figure 7:**
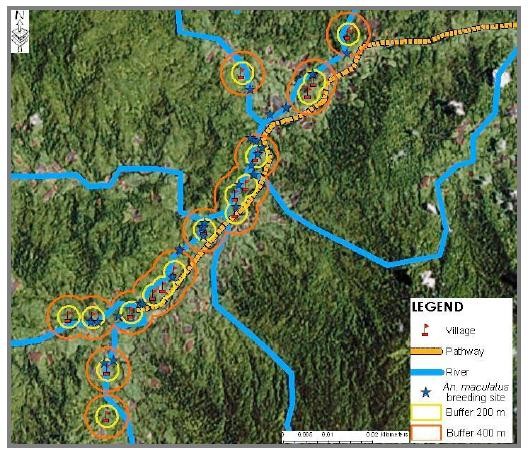
**Map of villages with 200 m and 400 m buffer zone for *An. maculatus *breeding sites**.

**Figure 8:**
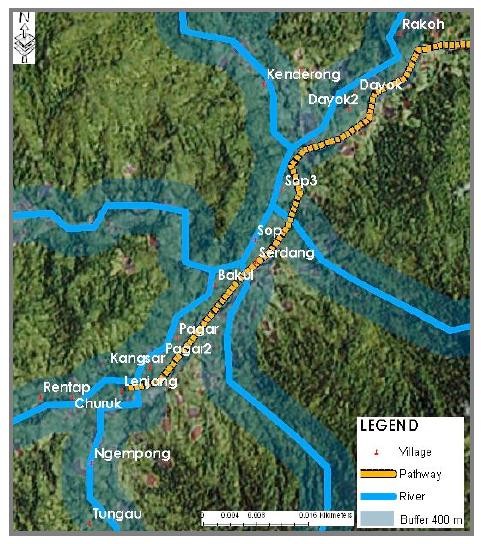
**Map of malaria transmission risk area in Pos Lenjang**.

With regards to *An. maculatus s.s*. breeding habitats, the study confirmed that not only the majority of these breeding habitats were located near to the river margin, but they were also located within the 200 m to 400 m range from the river to the nearest village. Figure 7 shows that all *An. maculatus *breeding habitats were found near to village vicinity. This map shows all affected villages in Pos Lenjang with 200 m and 400 m buffer zones and indicated that more than 80% of *An. maculatus *immature habitats were found in this zone. Based on this map, we could then made prediction on the malaria transmission risk area for Pos Lenjang. This prediction obviously would involve those areas within the 400 m buffer zone from river where the breeding sites were located to the nearest village situated along the river (Figure 8). This map illustrates that all 17 villages were in malaria risk transmission area.

## Discussion

Mosquitoes were quite discriminated in selecting sites for egg deposition. Although species overlapped in habitat preference, oviposition site selectivity was considerably species dependent [25]. The occurrence of different sibling species can explain part of the heterogeneity in behaviour. However, differences between individuals of the same species underlines the major role of environmental factors in determining the occurrence, distribution, seasonality, behaviour and vectorial statutes for each species [26]. In this study, spatial heterogeneity of anopheline and culicine mosquito species composition in Pos Lenjang, Kuala Lipis, Pahang was examined.

Environmental variables influence the suitability of aquatic habitats for anopheline and culicine larvae, but no significant association was found with the occurrence of both larvae genera. Studies on mosquito larval habitats were normally clustered along the river valleys or streams in hilly areas [7,27,28]. In this study, similar approach was applied in Pos Lenjang. River margins, waterfalls and stream pools were seen as potential habitats for development of anopheline and culicine larvae. Culicine larvae were found in all nine habitat groups suggesting that they are very versatile and highly adapted to different types of environment found in the sampling areas. Meanwhile, rock pools or water pockets with clear water form on the bank of rivers and waterfalls were the most common habitats associated with *An. maculatus s.s*. Larvae of *An. maculatus s.s*. were collected from various habitats either alone or in association with *An. macarthuri*.

A clear association was observed in the study areas between the distance to potential breeding sites and the variability in *An. maculatus s.s*. larvae. The study showed that *An. maculatus s.s*. was identified from breeding sources within 400 m from the nearest villages. A negative association was observed between the distribution of *An. maculatus s.s*. habitats and the distance to the nearest house, suggesting that *An. maculatus s.s*. prefer laying eggs in habitats near houses. Similar finding was reported by Rohani *et al *[7] in Pos Senderot, Pahang. Distance from house to breeding sites is the upmost factor to be considered for malaria risk mapping as the transmissions potential of vectors is maximized when oviposition habitat and humans are both available [29]. Previous studies found that the mosquito average flight range is influenced by time and the environmental condition, such as wind direction, wind current, physical barrier, hill, tree, distance from breeding sources and human habitat [30].

This study has determined the factors that influence the natural habitats of anopheline and culicine larvae. The ecology of the principal vector and other potential vectors were determined. This study also identified eleven species of mosquito breeding in nine habitat groups. In the case of low endemic malaria, rock pools with clear and muddy water should be inspected to identify vectors and suitable control measures should be applied. It was hoped that information on the abundance of mosquito species and other characteristics could be used to guide interventions to target larval control at specific sites or time periods. Furthermore, to achieve a satisfactory result, exhaustive targeting of all potential vector species is necessary.

Geographic Information System (GIS) and Remote Sensing (RS) are increasingly used for the study of spatial and temporal patterns of vector-borne diseases [31]. Kistemann *et al *[32] reported that RS is frequently applied on the investigation of the malaria risk area. The increasing number of successful applications of RS techniques to mosquito habitat mapping in North America provided sufficient foundation to transfer these approaches to satellite-borne sensor mapping of remote tropical areas, where both logistical and public health problems are significantly greater [33]. Cano *et al *[34] were able to define the spatial distribution of the vector-borne disease by the geographical distribution of the vectors and their vertebrate hosts.

Study by Sithiprasasna *et al *[11] suggested that classified remotely sensed data could potentially be used to estimate the distribution of immature and adult mosquito populations in the Republic of Korea. Thus, malaria control zone maps with the positions of villages in relation to roads, rivers, coastal anchorages and other topographical features can provide an important overview so that resources (manpower, vehicles and boats) can be allocated most efficiently to get the job done [35]. There are two correlated factors contributing to abundance of mosquito population in the study area; topographic features surrounding the villages and human density population for each village. Topographic features significantly affected the availability and stability of aquatic habitats [27,28,36]. Zhou *et al *[28] suggested that topography can have a significant impact on human malaria transmission through its effects on the spatial distribution of larval habitats and human settlement patterns. Generally, Pos Lenjang is characterized by hilly topography where the stagnant aquatic habitats at the bottom of the valley are formed by means of surface runoff from uphill, and from springs and groundwater seepage. Fewer studies have examined the possibility of predicting mosquito density on heterogeneous land cover. The data obtained in this study agreed with what has been observed by Roberts *et al *[37] in Belize, Central America, where by the population densities of mosquito (*Anopheles albimanus*) were much lower at houses located more than 1 km from rivers and marshes.

Immature stages of anopheline and culicine mosquitoes were found in a variety of aquatic habitats and under a variety of environmental conditions. However, larval survey is often very expensive, time consuming and burdensome to be carried out by Department of Health. As an alternative to this traditional ground-based survey approach of vector surveillance, application of RS data in a GIS to identify geographical characteristic that are correlated to vectors habitats was used. From a broader perspective, it would be applicable to apply the study method to other vector-borne diseases for which vectors are present in Pos Lenjang, Kuala Lipis, Pahang. This is the first essential step in assessing the risk of the re-emergence of malaria in Pos Lenjang, Kuala Lipis, Pahang.

## Conclusions

This study illustrates that the GIS based on the immature mosquito field information not only provided a graphical representation of the vectors population and their habitats, but also allowed generating a new variable, the distance to potential breeding grounds. Hence, combination of ground based sampling with modern RS technologies could be useful guidance for targeting vector borne diseases in specific localities.

## Competing interests

The authors declare that they have no competing interests.

## Authors' contributions

RA conceived the study and wrote the manuscript. WNW developed the study's GIS and conducted spatial and statistical analyses. ZI, RA, WNW, AAZ and MNI performed the field collections. ZI performed the identification. ZMN participated in drafting of the manuscript. LHL revised the manuscript and have given approval of the version to be published. All authors read and approved the final manuscript.
